# Reversible conversion between graphene nanosheets and graphene nanoscrolls at room temperature†

**DOI:** 10.1039/c8ra00475g

**Published:** 2018-03-09

**Authors:** Yanzhe Gai, Wucong Wang, Ding Xiao, Huijun Tan, Minyan Lin, Yaping Zhao

**Affiliations:** School of Chemistry and Chemical Engineering, Shanghai Jiao Tong University Shanghai 200240 P. R. China ypzhao@sjtu.edu.cn

## Abstract

In this paper, the reversible conversion between pristine graphene nanosheets and pristine graphene nanoscrolls at room temperature was reported. The graphene nanosheets were rolled up into the graphene nanoscrolls by silver nitrate in ethanol solution, and the fabricated graphene nanoscrolls were unfolded back to the graphene nanosheets in ethanol solution by adding ammonium hydroxide. The dynamic conversion state of the process was confirmed by the morphology of the intermediate samples captured using an optical microscope and scanning electron microscope. Also, AFM, TEM and Raman spectroscopy displayed that the graphene transformed from its nanoscrolls remained the structure and morphology of the started graphene. The reason for the formation of the nanoscrolls was assigned to the silver cyanide particles generated on the edge of the graphene. The freshly formed silver cyanide caused the unbalanced energy of the graphene surface by changing the pi electron distribution and triggered off the graphene to roll up. The unfolding of the graphene nanoscrolls back to the graphene nanosheets was attributed to the removal of the silver cyanide by the ammonia *via* forming the complex. This reversible conversion might be a novel and facile approach to make graphene nanoscrolls and to store the graphene. Also, it may bring new sight to the conversion research between two-dimension and one-dimension materials.

## Abstract 2

The reversible conversion between the pristine graphene nanosheets exfoliated from graphite and the pristine graphene nanoscrolls was achieved at room temperature.

## Graphical abstract

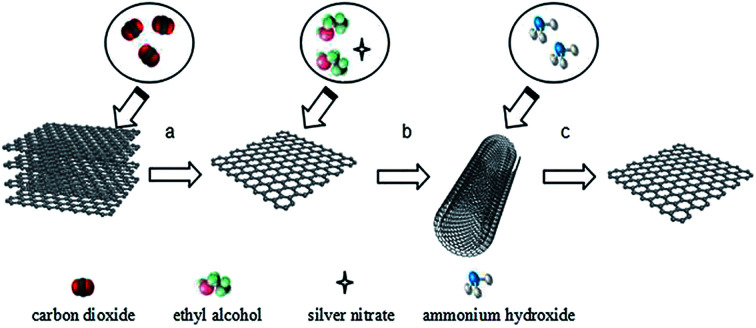

## Introduction

Graphene has attracted considerable attention since it was discovered because it has potential applications in numerous fields owing to its excellent electronic, mechanical, optical and thermal properties.^1–3^ It is a foundational material of the family of carbon nanomaterials, from which fullerene, carbon nanotube, and graphite can be formed from the structural point of view.^4–7^ Graphene nanoscrolls is a new type of carbon nanomaterial. It was first reported by Bacon in 1960 (called carbon nanoscrolls then), which was formed in a dcarc under a pressure of 92 atmospheres of argon and at 3900 K.^8^ Graphene nanoscrolls not only inherit graphene's superior properties but also have new excellent features. They can be useful candidates for microcircuit interconnects due to their high current density being sustained up to 5 × 10^7^ A cm^−2^;^9^ applied to supercapacitors, batteries, hydrogen storage, and catalyst supports due to their considerable surface area and highly porous structure;^10–12^ weaved as high-strength structural materials because they have a high degree of flexibility, tensile strengths up to 2000 kg mm^2^ and Young's modulus of more than 7 × 10^12^ dyne cm^−2^.^8^ Also, graphene nanoscrolls can be used as ion channels, controllable nano filters, tunable water channels and molecular sensors.^13–18^ Therefore, the preparation of graphene nanoscrolls has attracted increasing attention. Viculis *et al.* prepared graphene nanoscrolls^11^ through exfoliating graphite-intercalated with alkali metals at 200 °C under sonication with the high-energy of 500 W. Li J. L. *et al.* made graphene scrolls by high energy ball milling of graphite.^19^ Savoskin *et al.* obtained graphene nanoscrolls by using graphite-intercalation compounds and sonication.^20^ Xie reported a controlled way to prepare graphene nanoscrolls *via* rolling the monolayer graphene by evaporation of isopropyl alcohol solution.^9^ Quintana *et al.* rolled the exfoliated graphite sheets into graphene nanoscroll in dimethylformamide by using ferrocene aldehyde.^21^ Zheng J *et al.* reported the production of high-quality graphene nanoscrolls with microwave spark assistance in liquid nitrogen.^22^ Carotenuto G. *et al.* obtained nanoscrolls from graphite nanoplatelets by using a mechanical technique.^23^ Nevertheless, these methods can only produce a tiny amount of the graphene nanoscrolls, and the fabrication conditions are either harsh or difficult to control too. Therefore, the real practical application of the graphene nanoscrolls is sparsely reported. Notably, no articles on the reversible conversion between graphene nanosheets and graphene nanoscrolls have been published up to date.

In this letter, we reported a novel and facile approach to realize the mutual transformation of graphene nanosheets and graphene nanoscrolls by using silver salt and ammonia for the first time. The graphene nanoscrolls can be produced mainly from the graphene exfoliated from graphite and converted back to the graphene. SEM, TEM, AFM, XRD, and Raman spectroscopy were applied to characterize the morphology and structure of the graphene, the graphene nanoscrolls and the intermediate state of the conversion. The reversible conversion mechanism is also explored.

## Experiment section

### Materials

Graphite powder was purchased from Sinopharm Chemical Reagent Co., Ltd. Absolute ethanol (99.5%) and CO_2_ (99.5%) were obtained from the Shanghai Jiao Tong University chemical stores. Ammonium hydroxide (25–28%) and silver nitrate (99.8%) were purchased from Sinopharm Chemical Reagent Co., Ltd.

### Reversible conversion process of graphene and graphene nanoscrolls

#### Fabrication of graphene nanoscrolls from graphene

The graphene was firstly prepared by shear exfoliation in supercritical carbon dioxide based on our previously published article.^24^ Then, a certain amount of the as-prepared graphene was dispersed in ethanol solution into which a certain amount of silver nitrate was added forming the concentration of 0.01 mg ml^−1^. After stirring by a magnetic mixer for 30 min, the black sediment was formed and separated from the solution *via* vacuum filtration. The collected black sediment (named as graphene nanoscrolls) was dried in a vacuum oven for 24 h.

#### Conversion of graphene nanoscrolls into graphene

A certain amount of the as-prepared graphene nanoscrolls were firstly dispersed in 95% aqueous ethanol solution. Then, the ammonium solution (25–28%) was added dropwise into the ethanol solutions of the graphene nanoscrolls from which a small amount of solution was taken out at regular interval for characterization.

### Characterization

The morphology and the size of the samples were characterized by scanning electron microscopy (FE-SEM, Hitachi S-4800) on silicon substrates and were analyzed by transmission electron microscopy (TEM, JEOL JEM-2100F) on standard copper grids. The lateral size and thickness of samples were examined by atomic force microscopy (AFM, NanoNavi/E-Sweep, SII NanoTechnology, Inc.) on mica substrates. X-ray diffraction (XRD) patterns were carried out on a model of D/max-2200/PC X-ray diffractometer (XRD, Rigaku Co., Japan). The samples were irradiated at 10–70° with a scan rate of 5° min^−1^. Raman spectroscopy was measured on Renishaw inVia Reflex Raman System equipped with a 532 nm laser source.

## Results and discussion

**Fig. 1:**
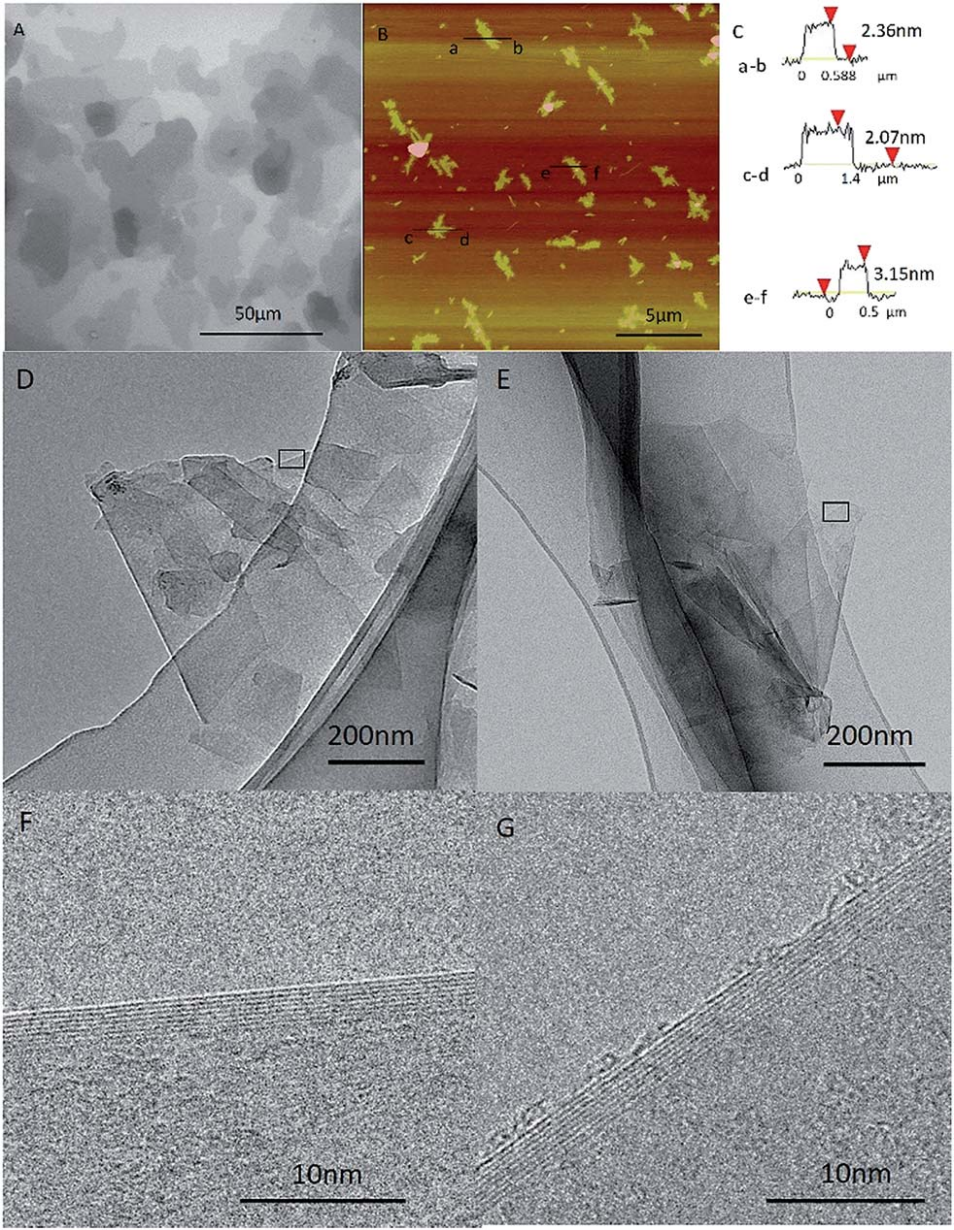
The characterization of the graphene made from graphite. (A) SEM image of graphene sheets, (B) AFM image with several graphene sheets, (C) height profiles of the graphene sheets obtained from positions indicated in (B), TEM and HRTEM images of the graphene: (D) and (E) exfoliated from graphite, (F) and (G) HRTEM images of the squares of (D) and (E) samples.

In order to compare the graphene made from the graphite (named as GMG) with the ones converted back from the graphene nanoscrolls (designated as GNS), we characterize the morphology and structure of the GMG using SEM, TEM, and AFM firstly. An SEM image (Fig. 1A) displays that the graphene sheets clustered together and tiled each other forming the map-like patterns.^25,26^ Their thickness and the lateral size can be identified from the AFM images. The average lateral dimension of the graphene nanosheets was around 1–5 μm as shown in Fig. 1B. The thickness of three specific pieces of the graphene nanosheets is 2–3 nm corresponding to the layer numbers of 5–9 layers as shown in Fig. 1C. TEM images exhibit that some individual graphene nanosheets adhered to each other (Fig. 1D), a piece of graphene sheet was folded (Fig. 1E), and the size of the graphene nanosheets was estimated to be about 1 μm. The HR-TEM images (Fig. 1F and G) indicate that the layer number of the graphene sheets is 7–8 layers, which is agreement with that of the AFM.

**Fig. 2:**
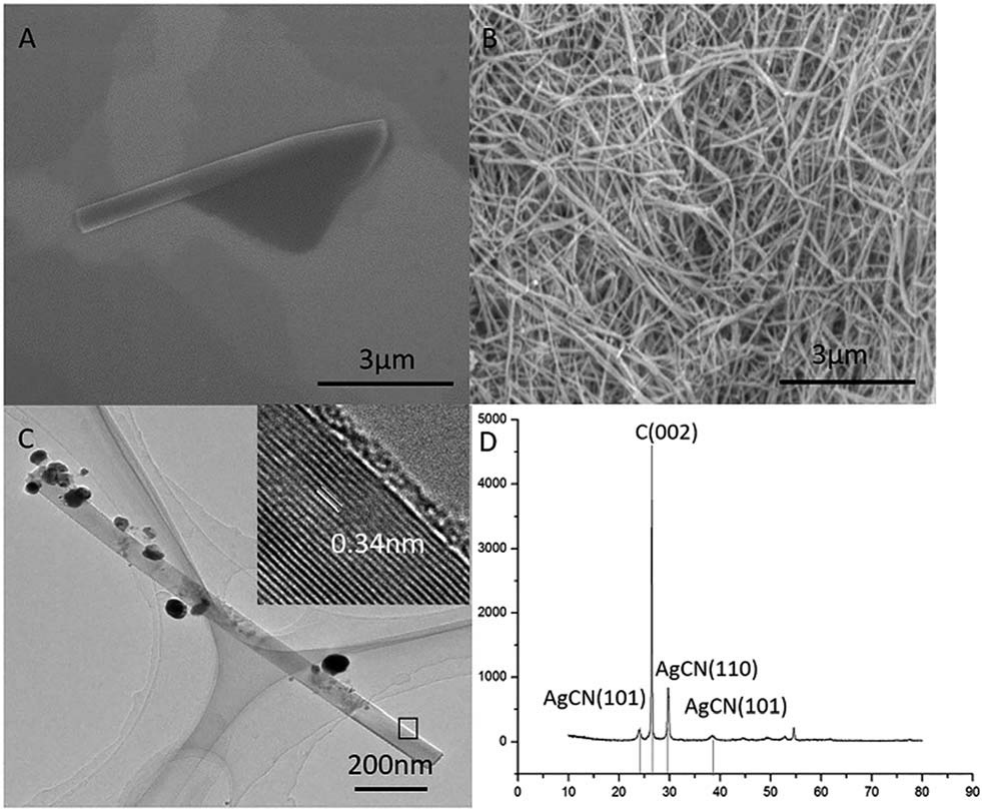
The characterization of the graphene nanoscrolls. (A) SEM image of an unfinished graphene nanoscroll, (B) SEM image of massive graphene nanoscrolls, (C) TEM and HRTEM image of a graphene nanoscroll, (D) XRD patterns of the graphene nanoscrolls.

Then, a certain amount of the as-prepared graphene was scrolled by a certain amount of silver nitrate in ethanol solution. We investigated the conversion of GMG into the GNS by applying SEM, TEM, and XRD, respectively. We can find an intermediate state of the isolated GNS from the SEM image (Fig. 2A), which has an open end. It implies that the conversion process was not finished. Accordingly, we can infer that this GNS was formed by scrolling starting from one edge of a GMG sheet and was pinned down until the end. Thus, the GMG was eventually converted into the GNS according to this process. In this way, a significant amount of GNS was formed as shown in Fig. 2B, from which it can be seen that the two-dimension GMG was disappeared. These GNS look like a cylindrical and fusiform structure and their diameter and length are about 80–150 nm and around several microns, respectively. Fig. 2C shows a typical TEM image of an individual GNS, from which it can be seen that it has a tubular-structure which looks like a multi-walled carbon nanotube. The interlayer spacing of the GNS is about 0.34 nm similar to that of graphite as shown in the inset of Fig. 2C. Fig. S3† verifies the structure of the GNSs too. The TEM image in Fig. S3a† indicates the tube-like structure of the specific GNS and the Fig. S3b† indicate the typical electron diffraction of the GNS. The diameter and the length of the GNS are approximately 60 nm and about 1.5 μm, respectively. This length falls within the size range of the GMG. Also, it can be seen that there are some nanoparticles on the surface of the GNS (Fig. 2C). We applied XRD to identify them. Fig. 2D displays the XRD patterns of the GNS including the nanoparticles. The sharp intensity peak at 2*θ* = 26.38° is attributed to the graphite crystallinity (002). The diffraction peaks at 2*θ* values of 24.03°, 29.74°, and 38.35° are assigned to the AgCN (101), AgCN (110) and AgCN (012) planes, respectively. Therefore, the nanoparticles formed on the surface of the GCN are identified to be AgCN. Also, the formed AgCN has been demonstrated using other methods.^27^ The role of the forming AgCN in the conversion of the GMG into the GNS will be explained in later.

**Fig. 3:**
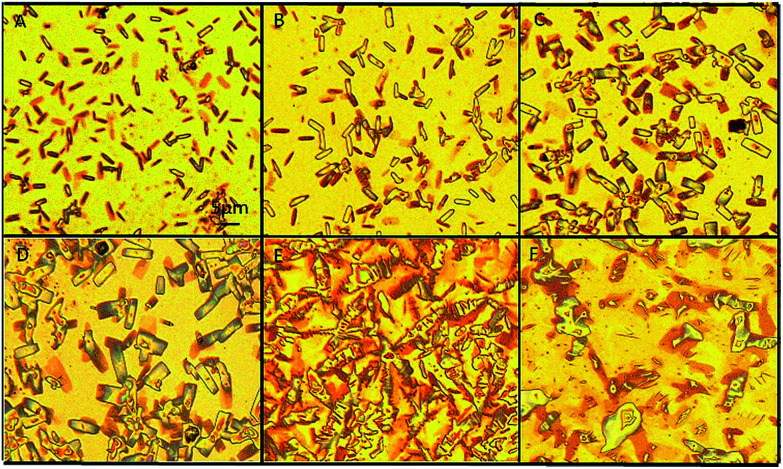
Optical microscope images of the graphene nanoscrolls collected at regular interval after adding aqueous ammonia. (A) 0 min, (B) 5 min, (C) 10 min, (D) 15 min, (E) 20 min, (F) the sample of (E) was treated in an ultrasonic water bath (120 W) for 20 min.

We found an interesting phenomenon when aqueous ammonium was added to the GNS ethanol solution to remove the AgCN: the GNS was converted back to the graphene sheets (named as GCG). To investigate this exciting phenomenon, we designed a series of experiments for the reaction of the GNS with the aqueous ammonium. The morphology and structure of the resultant samples obtained at a regular interval period were characterized. The images of the optical micrograph shown in Fig. 3 recorded the process of the morphology change of the GCN when 0.1 ml aqueous ammonium was added into 10 ml of the GNS ethanol solution (0.2 mg ml^−1^). It was observed by an optical microscope at an interval of five minutes until the GNS converted into the GCG completely. Fig. 3A shows the GNS evenly dispersed on the substrate after adding ammonia. The diameter of the GNS became bigger when the reaction was 5 min as shown in Fig. 3B. Some slender rectangles of the GNS can be seen, which suggests that it started loosening as soon as adding aqueous ammonium. With increasing the reaction time, the GNS become looser and looser as shown in Fig. 3B–D resulting in increasing its diameter. When the reaction time increased to 20 min, the GNS completely lost the nanoscroll feature becoming back the graphene sheets as shown in Fig. 3E. They possessed a lot of wrinkles and piled together on the substrate. At this stage, the GNS converted into the GCG. In order to spread further this wrinkled graphene into the graphene sheets thoroughly, we treated the sample in an ultrasonic water bath (120 W) for 20 min. We can see from Fig. 3F that the unfolded graphene nanosheets were formed eventually.

**Fig. 4:**
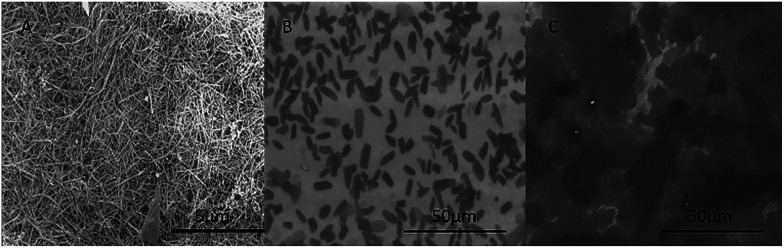
SEM images of the conversion of graphene nanoscrolls into graphene. (A) SEM image of the first graphene nanoscrolls, (B) SEM image of the intermediate state of the graphene nanoscrolls into the graphene, (C) the converted graphene from the graphene nanoscrolls.

Also, we captured the process of the samples during the conversion of the GNS into the GCG using SEM to observe the micro-process of the translation. Fig. 4A displays the SEM image of the first GNS. Fig. 4B showed the intermediate state of the GNS converting to GCG when 0.1 ml aqueous ammonium was added to 10 ml of the GNS ethanol solution (0.2 mg ml^−1^) for 10 min. Fig. 4C displays the unfolded graphene which looks similar to the GMG as shown in Fig. 2A. The morphology of the GCG seems identical with the GMG, which means the GNS converted entirely back to the graphene.

To confirm the point aforementioned, we applied TEM, AFM, TEM and Raman spectra to characterize the morphology and quality of the GCG. TEM images (Fig. S1A and B†) indicate that the GCG is clustered together and is similar to the GMG, and their lateral sizes are about 1–4 μm. The edges of the graphene shown at high magnification TEM as shown in Fig. S1C and D† reveal that the layer number of the graphene sheets is 7. AFM images indicate that the GCG is about 3–5 μm in size and 3.05 nm and 2.53 nm in thickness as shown in Fig. S1E–G.† It suggests that the graphene sheets are about 6–8 layers, which is almost the same as the GMG.

**Fig. 5:**
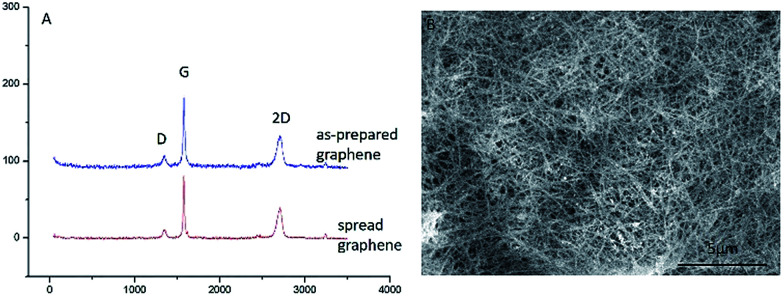
Raman spectra of graphene and SEM image of CNSs. (A) Raman spectra of the graphene made from graphite and converted from the graphene nanoscrolls, (B) SEM image of CNSs stored for six months.

Fig. 5A shows the Raman spectra of the GMG and the GCG. It can be seen that the intensity and the position of the D peak of the GCG are as the same as the GMG. It suggests that the structure of the graphene was not damaged during the mutual conversion between the graphene and graphene nanoscrolls. The graphene remained high quality during the reversible change process. Also, the graphene nanoscrolls have excellent stability. It can maintain its original appearance even it was stored for more than six months as shown in Fig. 5B. Also, we can infer that the yield of both the graphene nanoscrolls and the converted back graphene is almost 100% because there are no graphene sheets found in the Fig. S2A and B† and no graphene nanoscrolls found in the Fig. S2C and D.† We can conclude that the reversible conversion of the graphene and the graphene nanoscroll has been achieved. It implies that the reversible conversion process can be an excellent way to store graphene nanosheets as it would not change the original morphology and quality of the graphene. It is still a challenging issue to store graphene because it is prone to agglomerate without surfactant or organic reagent resulting in inconvenient practical applications. Therefore, this approach achieving the reversible conversion process can not only produce graphene nanoscrolls largely but also be an efficient way to store graphene.

**Fig. 6:**
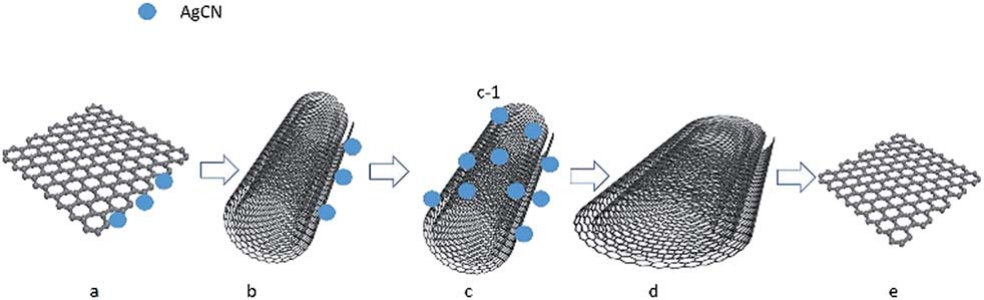
Schematic route of the reversible conversion between the graphene and graphene nanoscrolls. (a) Initial graphene on which the silver cyanide nanoparticles were adsorbed, (b) intermediate scrolled-graphene (c) formed graphene nanoscroll, (d) loose graphene nanoscroll, (e) converted graphene.

The interconversion of the pristine graphene and the pristine graphene nanoscrolls is reported for the first time. We proposed a mechanism to explain this process. As shown in Fig. 6, the silver cyanide nanoparticles generated in the mixture of the graphene sheets and the ethanol solution of silver nitrate trigger the started graphene (Fig. 6a) to scroll into the graphene nanoscroll (Fig. 6c) *via* the intermediate state (Fig. 6b). The competence of the bending energy of the graphene and the van der Waals forces between the graphene sheets decide if the former could become the latter. We think the generated-AgCN^27^ triggers the curling of the graphene, which overcomes the stain energy barrier when the graphene starts to bend. Upon the overlap of the graphene sheet occurs, the van der Waals force between the graphene sheets is more significant than the bending force of the graphene, which resulting in the curling process to continue until reaching the stable state of the nanoscrolls. The silver cyanide nanoparticles might be generated much more at one edge of the graphene but scarce in the other sides because the reaction was at random. Then, the unbalanced energy of the graphene surface was generated by the formed silver cyanide nanoparticles through changing the pi electron distribution of the graphene sheet because of silver ion attraction like the isopropyl alcohol causing a surface strain in graphene by immersion.^29^ This unbalanced surface strain caused the opposite edge of the graphene sheet to start to bend. Upon the surface strain is big enough to overcome the energy barrier of the graphene bending, the graphene sheet would begin to roll until reaching the stable state, a graphene nanoscroll, by van der Waals forces. This speculation is proved by the fact that the silver cyanide nanoparticles were on the surface of the scrolls but not trapped in the scroll structure as shown in Fig. 2C. In the contrast process, the silver cyanide nanoparticles were dissolved in the solution *via* reacting with the ammonium ion when the aqueous ammonium was dropwise into the solution. With the dissolving of the silver cyanide nanoparticles, the spreading force of the graphene nanoscrolls was big enough to make itself loosen gradually (Fig. 6d) and finally to be converted to the graphene (Fig. 6e). In the published paper,^28^ Sharifi *et al.* claimed that the scrolling of the nitrogen-doped reduced graphene oxide sheets into the nanoscrolls was initiated by the strong adsorption of the maghemite nanoparticles at nitrogen defects in the graphene lattice and their mutual magnetic interaction, and the nanoscrolls could be returned to half-open structures and open sheets fully upon removal of the maghemite nanoparticles by acid treatment in different period. Therefore, we think the mechanism of the scrolling and unwinding in our work were similar to the Sharifi's ones regarding the rolling and unrolling caused by forming nanoparticles and removing nanoparticles. However, the role of the silver cyanide nanoparticles in initiating curling in our work might be different because the silver cyanide does not have magnetic properties, which suggests that the nanoparticles without magnetic properties can cause scrolling too if the conditions are suitable. Also, the system in the published paper^28^ was complicated, while our scrolling system is straightforward.

## Conclusions

A novel and facile approach has been developed to achieve the reversible conversion between graphene nanosheets and graphene nanoscrolls. We have demonstrated the products of the mutual transformation by using an optical microscope, SEM, TEM, and AFM and Raman spectra, respectively. The graphene converted from the graphene nanoscrolls remained its initial structure and morphology. The silver cyanide at the edge of the graphene played essential roles in the transformation of the graphene into the graphene nanoscrolls and the reverse. The approach is expected to be useful in making graphene nanoscrolls and storing graphene.

## Conflicts of interest

There are no conflicts to declare.

## Supplementary Material

RA-008-C8RA00475G-s001
