# Supplementary material for: Reversible conversion between graphene nanosheets and graphene nanoscrolls at room temperature
Source: RSC Adv. 2018 Mar 9;8(18):9749–53. doi: 10.1039/c8ra00475g (PMC9078727; doi:10.1039/c8ra00475g)
Supplement: RA-008-C8RA00475G-s001 [file RA-008-C8RA00475G-s001.pdf]

Electronic Supplementary Material (ESI) for RSC Advances.
This journal is © The Royal Society of Chemistry 2018

Supporting Information for

Reversible conversion between graphene nanosheets and graphene nanoscrolls at room temperature

Yanzhe Gai, Wucong Wang, Ding Xiao, Huijun Tan, Minyan Lin, Yaping Zhao*

School of Chemistry and Chemical Engineering, Shanghai Jiao Tong University

Shanghai 200240, P. R. China

Corresponding author (E-mail:ypzhao@sjtu.edu.cn)

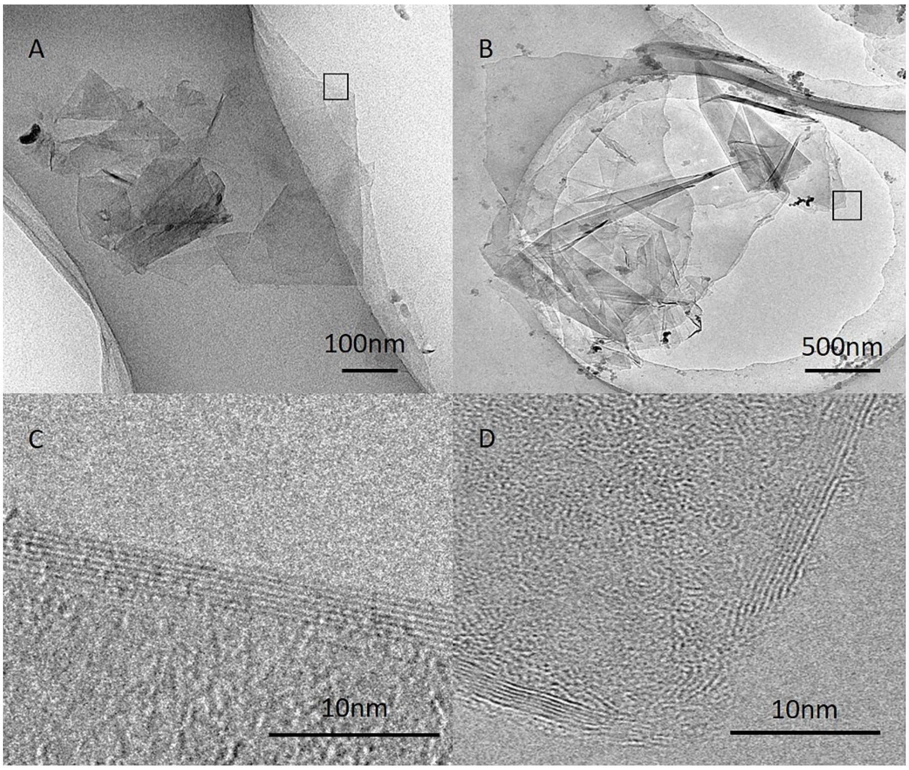

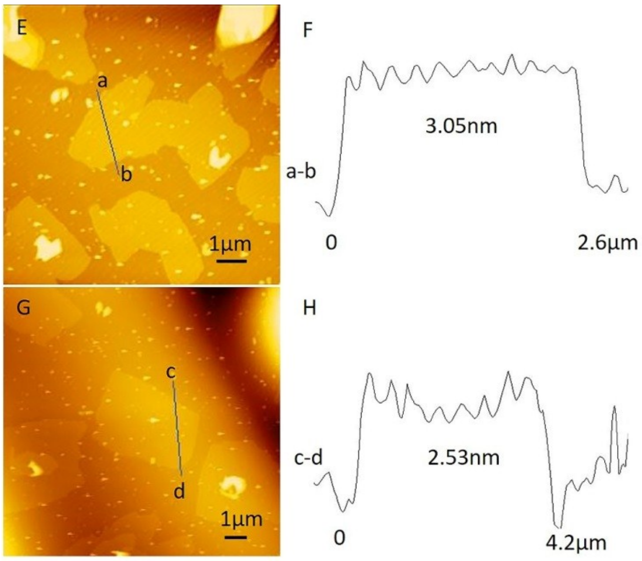

Fig.S1. The characterization of the graphene converted back from the graphene nanoscrolls. TEM and HRTEM images of the graphene: (A) and (B) converted back from the graphene nanoscrolls, (C) and (D) HRTEM images of the squares of (A) and (B) samples. (E) and (G) AFM images with several graphene sheets, (F) and (H) Height profiles obtained from positions indicated in (E) and (G).

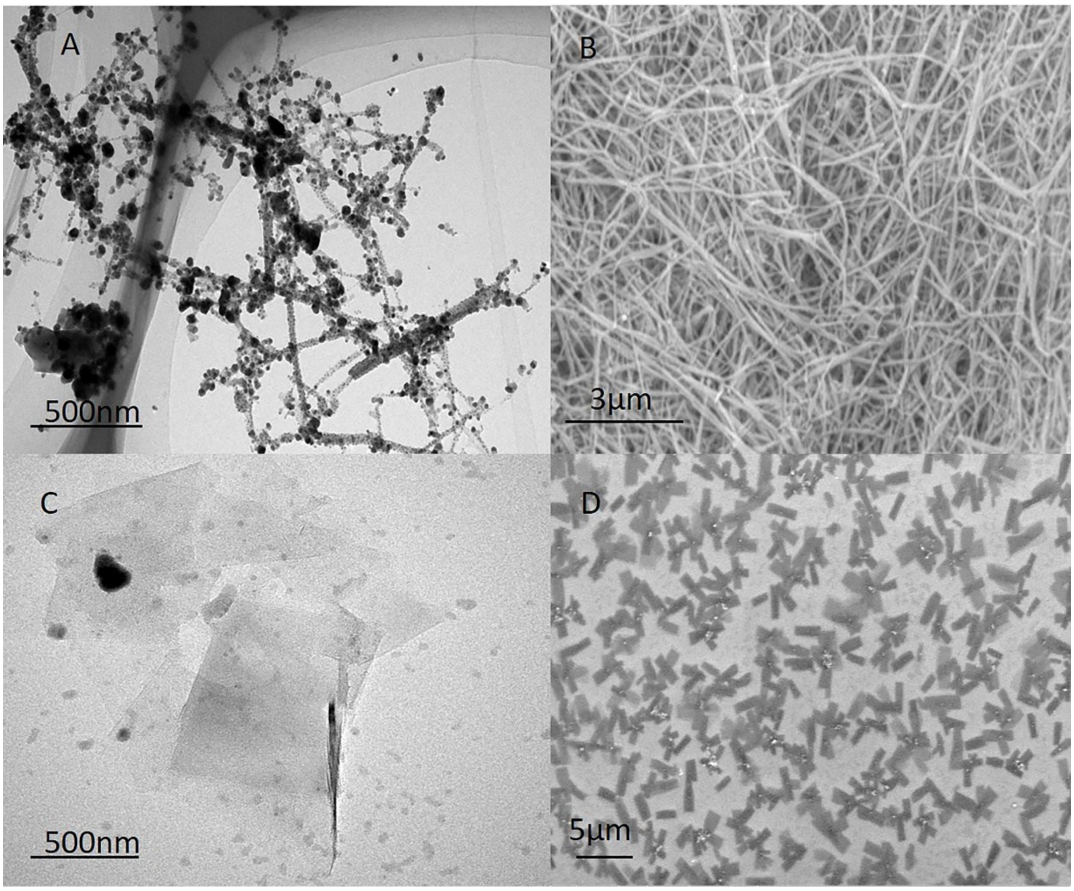

Fig.S2. The characterization of the graphene nanoscrolls and the graphene cluster converted from the graphene nanoscrolls**.** (A) and (B) TEM and SEM images of the graphene nanoscrolls cluster, (C) and (D) TEM and SEM images of the graphene cluster converted from the graphene nanoscrolls.

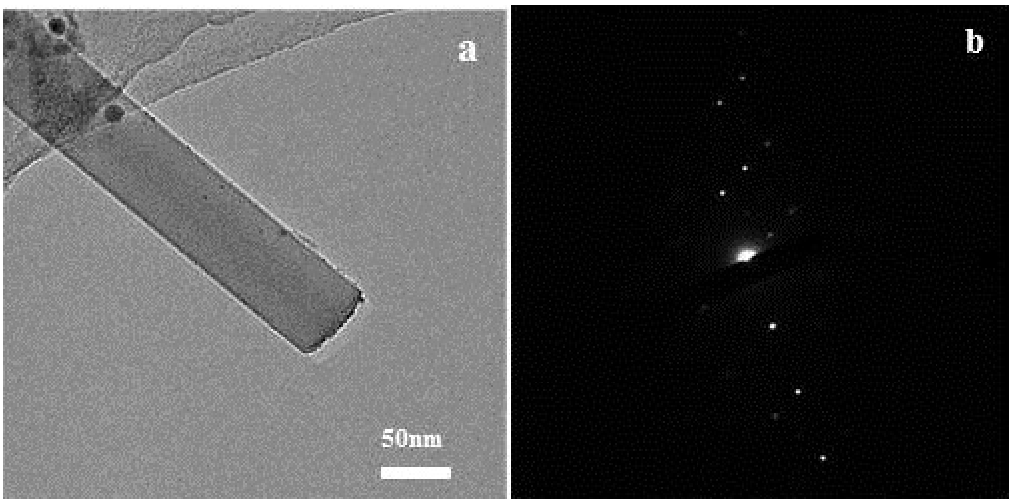

Fig.S3. High-magnification TEM image of the GNS (a) and its typical electron diffraction pattern (b).
